# Clascoterone in the Treatment of Acne Vulgaris: A Narrative Review

**DOI:** 10.3390/ph19081304

**Published:** 2026-08-18

**Authors:** Sara Shefa, Jagoda Szwach, Anna Karwowska, Aleksandra Wojno, Magdalena Łyko

**Affiliations:** 1 Student Research Group of Experimental Dermatology, University Centre of General Dermatology and Oncodermatology, Wroclaw Medical University, Borowska 213, 50-556 Wroclaw, Polandanna.karwowska@student.umw.edu.pl (A.K.); aleksandra.wojno@student.umw.edu.pl (A.W.); 2 University Centre of General Dermatology and Oncodermatology, Wroclaw Medical University, Borowska 213, 50-556 Wroclaw, Poland

**Keywords:** clascoterone, acne vulgaris, acne, cortexolone 17α-propionate, androgen receptor inhibitor, topical antiandrogen

## Abstract

Acne vulgaris is one of the most prevalent dermatological conditions, affecting up to 85% of individuals aged 11–30 years. Clascoterone cream is the first topical androgen receptor inhibitor approved by the U.S. Food and Drug Administration (FDA) for the treatment of acne vulgaris in patients aged 12 years and older, and the first agent in this class approved for use in both male and female patients. The aim of this narrative review is to summarize the current evidence regarding the efficacy, safety profile, and potential future applications of clascoterone in the treatment of acne vulgaris. A comprehensive literature search was conducted in PubMed/MEDLINE and ClinicalTrials.gov databases up to April 2026. Search terms included “clascoterone,” “cortexolone 17α-propionate,” and “androgen receptor inhibitor acne.” Eligible publications included randomized controlled trials, meta-analyses, open-label extension studies, retrospective analyses, case reports, and relevant review articles published in English. Clinical trial registries and regulatory agency announcements were also searched for ongoing studies and recent approval decisions. Phase 3 trials and meta-analyses demonstrate that twice-daily application of clascoterone 1% cream significantly reduces inflammatory and non-inflammatory lesion counts compared to vehicle, with efficacy sustained up to 12 months of treatment. The safety profile is favorable, with adverse events limited predominantly to mild and transient local skin reactions. Subgroup analyses have shown no significant sex-related differences in efficacy or safety, and the safety profile appears comparable between adolescents and adults. In an exploratory pooled subgroup analysis, lesion-count reductions were greater in adults than in adolescents, whereas the adjusted between-group comparison of IGA success was not statistically significant. Because no formal treatment-by-age interaction test was reported, these findings should be considered hypothesis-generating. Emerging but still incomplete data suggest potential applications in androgenetic alopecia and mild hidradenitis suppurativa. Clascoterone represents a novel addition to the therapeutic armamentarium for acne vulgaris, offering a unique mechanism of action with a favorable safety profile. Further research is warranted to establish its role in combination therapies and in other androgen-dependent dermatoses.

## 1. Introduction

Acne vulgaris is among the most prevalent and well-characterized dermatological conditions. It manifests through four primary lesion types: inflammatory lesions, including papules and pustules; non-inflammatory lesions, such as open and closed comedones; seborrhea; and varying degrees of scarring [1,2]. The condition affects up to 85% of individuals between the ages of 11 and 30, underscoring its high prevalence in the general population [3,4,5].

The pathophysiology of acne vulgaris is multifactorial and involves several interrelated mechanisms, including hormonal fluctuations, hyperactivity of the sebaceous glands, abnormal follicular keratinization, and alterations in the skin microbiome associated with *Cutibacterium acnes*. These processes contribute to immune system activation and subsequent cutaneous inflammation [6]. Other potential risk factors for disease severity remain under investigation and include genetic predisposition, socioeconomic status, dietary habits, personal hygiene, smoking, and psychological stress [7,8,9,10,11,12].

Given the wide spectrum of lesions observed in acne vulgaris, numerous therapeutic options are available, each with distinct mechanisms of action. In mild to moderate cases, topical agents such as benzoyl peroxide, retinoids, antibiotics, and azelaic acid are commonly employed. In more severe presentations, systemic therapies are indicated, including oral antibiotics, isotretinoin, hormonal therapy, and spironolactone. This restriction specifically concerns systemic antiandrogen therapy for acne and should not be extrapolated to androgen-modulating treatments used for other indications in men, such as 5α-reductase inhibitors for androgenetic alopecia. Clascoterone itself was developed for topical administration; oral clascoterone has not been established or approved as a treatment for acne vulgaris. Laser therapy has also demonstrated efficacy as an adjunctive treatment modality [1,2,13,14]. The management of acne is inherently complex and necessitates an individualized approach tailored to the patient’s specific clinical presentation and the underlying pathophysiological mechanisms.

Clascoterone is a novel therapeutic agent introduced to the pharmaceutical market with a distinct mechanism of action. In August 2020, it received official approval from the U.S. Food and Drug Administration (FDA) for the treatment of acne vulgaris in individuals aged 12 years and older. It is the first androgen receptor inhibitor approved for topical use in acne therapy, exerting its effect locally without inducing systemic hormonal alterations. Notably, it is also the first agent in this pharmacological class approved for use in both female and male patients [15,16,17,18,19].

The aim of this review is to summarize the current evidence regarding the use of clascoterone in the treatment of acne vulgaris. It outlines the drug’s efficacy, safety profile, and potential future applications. By consolidating existing data and highlighting areas in need of further investigation, this review seeks to enhance understanding of clascoterone’s role in clinical dermatology.

## 2. Materials and Methods

A comprehensive literature search was conducted using PubMed/MEDLINE and ClinicalTrials.gov databases. The search was performed up to April 2026 using the following terms: “clascoterone,” “cortexolone 17α-propionate,” and “androgen receptor inhibitor acne.” Boolean operators (AND, OR) were used to combine search terms as appropriate. In addition, European Medicines Agency and U.S. Food and Drug Administration regulatory pages, as well as manufacturer announcements, were screened for the most recent approval-related information and topline trial data.

Eligible publications included randomized controlled trials, meta-analyses, systematic reviews, open-label extension studies, retrospective analyses, case reports, and relevant narrative review articles published in English. Clinical trial registries were also searched for ongoing and recently completed studies. Reference lists of included articles were manually screened for additional relevant publications. Two recent peer-reviewed narrative reviews on topical clascoterone for acne vulgaris were identified during the search [20,21]; their findings were considered in the synthesis, although neither incorporated the most recent regulatory approvals and phase 3 androgenetic alopecia trial results that are summarized in the present work. No formal risk-of-bias assessment was performed, consistent with the narrative review design of this work.

## 3. Mechanism of Action

Excessive sebum production is a principal factor contributing to the pathogenesis of acne vulgaris. Sebum synthesis is stimulated by the binding of androgens, including testosterone and dihydrotestosterone (DHT), to androgen receptors (AR), which are expressed throughout the skin, notably within sebaceous glands, sebocytes, and dermal papilla cells [22,23,24].

**Figure 1:**
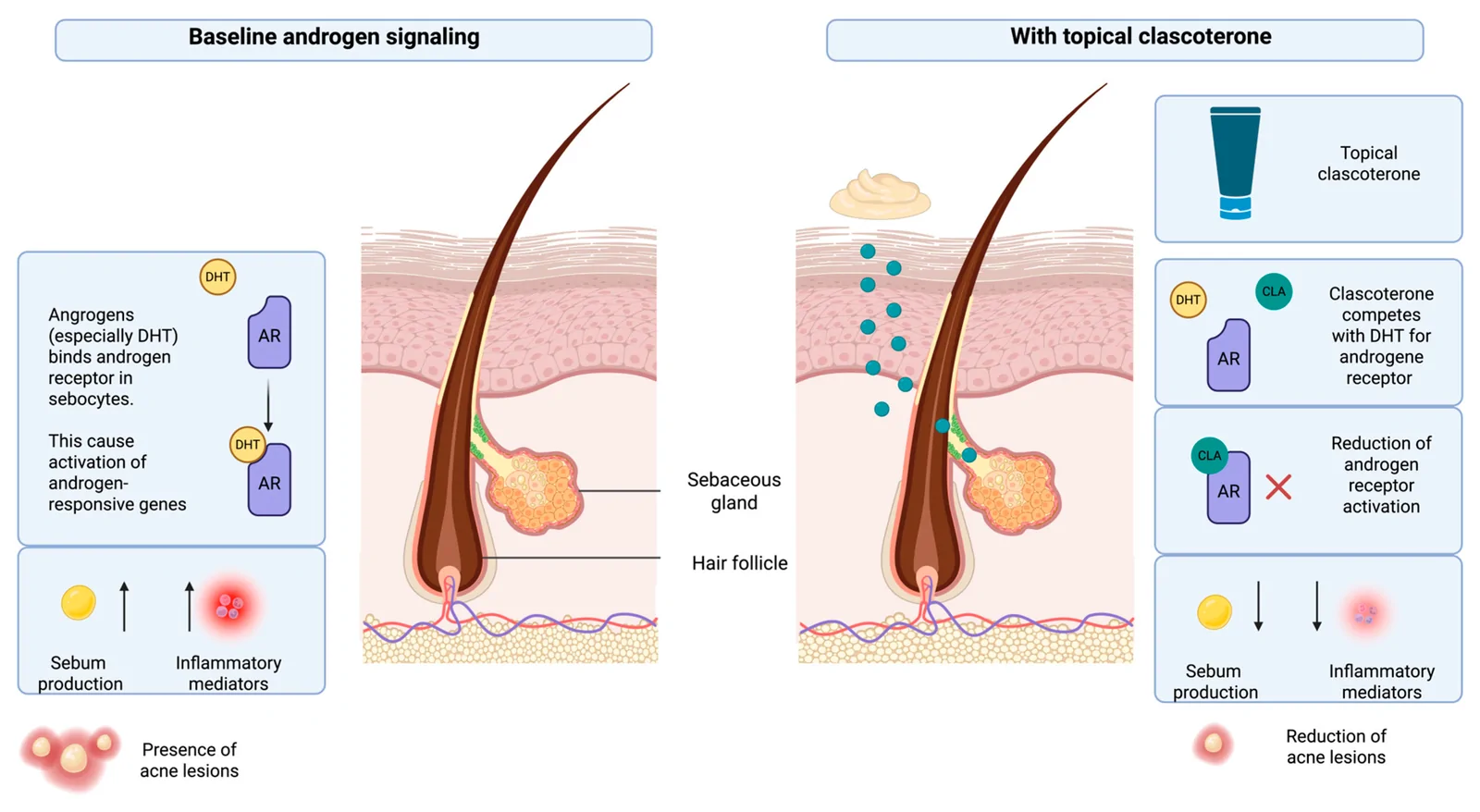
Proposed mechanism of action of topical clascoterone in acne. Clascoterone is thought to inhibit androgen receptor signaling in sebocytes by competing with DHT for receptor binding, leading to reduced sebum production, decreased inflammatory signaling, and improvement of acne lesions. Created in BioRender. Łyko, Magdalena. (2026) https://BioRender.com/cpa4mcp.

Clascoterone 1% cream (cortexolone 17α-propionate) functions as an androgen receptor inhibitor. Its mechanism of action involves the competitive inhibition of androgen-mediated stimulation of sebaceous glands, thereby reducing sebum secretion and subsequent acne development [15]. By binding to androgen receptors in a manner competitive with DHT, clascoterone blocks the downstream signaling that leads to increased sebocyte proliferation and lipogenesis. This mechanism distinguishes clascoterone from other available topical acne therapies, which target bacterial colonization, keratinization, or inflammation, but do not directly address the hormonal component of acne pathogenesis. (Figure 1).

Topical administration of clascoterone enables selective targeting of cutaneous androgen receptors, while its rapid metabolic inactivation to cortexolone—an inactive metabolite—minimizes the potential for systemic adverse effects [16]. Unlike systemic antiandrogen therapies used for acne such as spironolactone, which carry risks of systemic hormonal effects and are generally unsuitable or not recommended for male patients, clascoterone’s local mechanism of action allows its use in both sexes without systemic antiandrogenic consequences [15,25].

## 4. Efficacy

The efficacy of clascoterone 1% has been confirmed, among others, in a pooled analysis of two randomized phase 3 trials in patients aged ≥12 years with acne vulgaris. After 12 weeks, the proportion of “IGA success” (achieving an IGA score of 0 (clear) or 1 (almost clear) together with at least a 2-grade improvement from baseline) was 19.9% in the treatment group compared to 7.7% in the placebo group (*p* < 0.0001). Greater reductions in lesion counts were also observed with clascoterone compared with vehicle, with least-squares mean absolute changes from baseline of −20.8 vs. −11.9 for non-inflammatory lesions (corresponding percentage changes from baseline of −30.8% vs. −18.3%; *p* < 0.0001), −19.7 vs. −14.0 for inflammatory lesions (−46.2% vs. −32.5%; *p* < 0.0001), and −40.0 vs. −26.1 for total lesions (−37.8% vs. −25.1%; *p* < 0.0001) [26]. Measures of dispersion were not reported for these pooled estimates; in the two underlying pivotal trials, however, the between-group differences in absolute lesion-count change ranged from −3.8 to −8.6 lesions for inflammatory and non-inflammatory lesions, with 95% confidence intervals excluding zero in all comparisons [18]. Another study demonstrated that after 12 weeks of twice-daily application, sebum production decreased by 27%, inflammatory lesions by 54%, and non-inflammatory lesions by 34%, with statistically significant reductions observed from week 2 for non-inflammatory lesions, week 4 for inflammatory lesions, and week 6 for sebum measurements [27]. Importantly, efficacy was independent of sex, distinguishing clascoterone from oral antiandrogen therapies that are restricted in men [25]. Furthermore, an analysis of both short-term and long-term clinical trials showed that the efficacy of clascoterone 1% is not only sustained but also increases with continued treatment for up to 12 months [28,29,30].

The frequency of clascoterone 1% application is also of importance. It has been shown that twice-daily (BID) use of clascoterone 1% is associated with greater reductions in both inflammatory and non-inflammatory lesions compared with once-daily regimens [31]. This finding was further confirmed in a meta-analysis including data from over 2000 patients, demonstrating superiority over placebo not only in reducing inflammatory and non-inflammatory lesions but also in the proportion of patients achieving “IGA success” [32].

Indirect evidence from systematic reviews and network meta-analyses suggests that no statistically significant differences in efficacy were observed between clascoterone and selected topical retinoids, including trifarotene and tazarotene, although direct head-to-head randomized trials are lacking [33,34]. Clascoterone differs mechanistically from retinoids and benzoyl peroxide by targeting cutaneous androgen receptor signaling. Its predominantly local adverse-event profile may be advantageous in patients for whom systemic antiandrogen therapy is unsuitable or undesirable. A network meta-analysis also indirectly compared topical clascoterone with oral spironolactone; however, differences in study populations, outcome definitions, and trial designs preclude firm conclusions regarding their relative efficacy [32]. Thus, clascoterone should currently be viewed as an additional treatment option rather than as a proven substitute for established topical or systemic therapies.

It should also be emphasized that clascoterone is often used in combination therapy with topical retinoids, benzoyl peroxide, and antibiotics. In a small pilot study (n = 6), patients received clascoterone 1% cream (BID) together with a gel containing clindamycin 1.2%, adapalene 0.15%, benzoyl peroxide 3.1% (QD) for 8 weeks. No new tolerability issues were identified, and the observed adverse events, such as burning or erythema, were mild and did not significantly affect adherence [35]. It should be noted, however, that the very small sample size of this study limits the generalizability of these findings. Furthermore, both Canadian and real-world data indicate that clascoterone 1% is effective and well tolerated, whether used as monotherapy, adjunctive treatment, or maintenance therapy, with more than half of patients continuing therapy for longer than 3 months [30,36].

In a pooled post hoc analysis of 1421 patients aged ≥12 years from two randomized, double-blind, vehicle-controlled phase III trials, the efficacy of clascoterone 1% cream applied twice daily for 12 weeks was compared between adolescents aged 12–17 years and adults aged ≥18 years [37]. Investigator’s Global Assessment (IGA) treatment success, defined as an IGA score of 0 or 1 together with a ≥2-grade improvement from baseline, was evaluated using logistic regression adjusted for study, demographic characteristics, and baseline IGA, whereas lesion-count changes were assessed by analysis of covariance. IGA success was achieved by 16.4% of clascoterone-treated adolescents versus 3.9% receiving vehicle (OR = 4.70; 95% CI: 2.48–8.91; *p* < 0.001) and by 23.3% of clascoterone-treated adults versus 9.4% receiving vehicle (OR = 3.06; 95% CI: 1.93–4.85; *p* < 0.001). Although the crude response rate was 6.9 percentage points higher in adults, the adjusted age-group comparison was not statistically significant (OR for adolescents versus adults = 0.77; 95% CI: 0.50–1.17; *p* > 0.05). Adults nevertheless showed significantly greater reductions in inflammatory lesions by 3.62 lesions (95% CI: 1.05–6.19; *p* = 0.006), non-inflammatory lesions by 9.06 lesions (95% CI: 4.50–13.62; *p* < 0.001), and total lesions by 13.40 lesions (95% CI: 7.16–19.64; *p* < 0.001) compared with adolescents. The authors indicated that these differences may reflect type I error and proposed lower treatment adherence in younger patients and greater androgenic activity during puberty as possible explanations [37]. The adherence hypothesis is supported by observational evidence showing an independent association between younger age and poorer adherence to acne treatment [38], whereas the mechanistic hypothesis is consistent with in vitro findings that clascoterone binds the androgen receptor and inhibits androgen-regulated lipid and inflammatory cytokine production in human sebocytes [39]. However, adherence and androgen concentrations were not compared between age groups, and no formal treatment-by-age interaction test was reported. Therefore, the lesion-count differences should be interpreted as exploratory rather than definitive evidence of greater intrinsic efficacy in adults [37].

The potential efficacy of clascoterone has also been suggested in other androgen-dependent dermatoses, including mild forms of hidradenitis suppurativa, where 83% of patients demonstrated improvement after 12 weeks of treatment without significant adverse events [40].

## 5. Adverse Events and Safety Profile

Assessment of adverse events and overall safety is a crucial aspect of evaluating clascoterone for the treatment of acne vulgaris.

### 5.1. Clinical Trial Data and Subgroup Analysis

Two multicenter, double-blind, phase III studies, including 1440 patients and lasting 12 weeks, evaluated the safety of clascoterone. Patients were randomly assigned to receive either clascoterone 1% cream or vehicle cream. The use of clascoterone demonstrated favorable efficacy and a good safety profile with low adverse event rates. The most common treatment-emergent adverse events (TEAEs) were nasopharyngitis, headache, and oropharyngeal pain. Severe adverse events, including pneumonia and contusion, were observed only in the vehicle group and did not occur in patients treated with clascoterone. The most frequently observed local skin reactions were erythema (clascoterone: 11–13%, vehicle: 14–16%), scaling/dryness (clascoterone: 9–12%, vehicle: 7–13%), and pruritus (clascoterone: 5%, vehicle: 5–6%) [18].

Furthermore, subgroup analyses suggest a comparable safety profile of clascoterone cream 1% across age and sex strata. The frequency of TEAEs was almost identical to the vehicle across adolescent (10.8%), adult (11.5%), male (9.8%), and female (11.9%) patients. The vast majority of these events were mild or moderate in severity, and no serious TEAEs were reported in the group using clascoterone [37].

Another study evaluated the safety of twice-daily clascoterone use over a 9-month period in patients with acne vulgaris. Similar rates of TEAEs were reported between clascoterone and vehicle groups (18.3% vs. 17.9%), with nasopharyngitis and mild local reactions being the most common. Rare events included acne exacerbation and a single case of hair depigmentation within the treatment area, which led to treatment discontinuation [17].

### 5.2. Combination Therapy and Systemic Safety

A recent 12-week study of clascoterone reported no treatment-related adverse events, with both investigator and patient assessments indicating good tolerability. Mild local reactions such as dryness, redness, or burning were not observed or rated as minimal [27].

A prospective pilot evaluation study aimed to assess the efficacy of combination therapy with clascoterone and a triple-combination medication containing clindamycin, benzoyl peroxide, and adapalene, as well as the potential to target all four major components of acne pathogenesis. The study demonstrated that clascoterone can be safely used in combined treatment, with adverse events limited to mild local reactions [35]. Similarly, a retrospective study has shown favorable results for the combination of oral isotretinoin and clascoterone, demonstrating a good safety profile and improved efficacy. This suggests that clascoterone may be safely combined with other standard acne treatments to optimize therapeutic outcomes [41].

A phase 1 study investigating the effects of clascoterone on the QT interval confirmed its cardiovascular safety [42]. In addition, clinical data indicate that clascoterone does not cause clinically significant disturbances in blood potassium levels, so routine laboratory monitoring is not required in patients within the approved age range [43]. Laboratory analysis (n = 600) showed that the incidence of elevated potassium values was comparable between the clascoterone group (5.3%) and placebo (3.9%). These episodes were not considered adverse events and did not lead to treatment discontinuation, indicating no clinically relevant risk of hyperkalemia with clascoterone therapy [43]. Long-term studies also show no systemic antiandrogenic effects of clascoterone, such as decreased libido or feminization in male patients [17].

Phase 1 studies further indicated minimal systemic exposure to clascoterone (<1% of the administered dose excreted in urine, mean plasma concentrations 0.23–2.71 ng/mL), no accumulation after 42 days of treatment, and no clinically significant irritant or sensitizing potential [44]. However, under maximum-use conditions (well above the recommended dose), clascoterone may cause transient, subclinical suppression of the hypothalamic–pituitary–adrenal (HPA) axis, with normalization within approximately 4 weeks [45]. Under maximum-use conditions, in which clascoterone was applied at doses exceeding the approved regimen, transient laboratory evidence of HPA-axis suppression was observed in 1 of 20 adults (5.0%) and 2 of 22 adolescents aged 12 to <18 years (9.1%), corresponding to 3 of 42 participants aged ≥12 years (7.1%) [45]. HPA-axis suppression was defined as a 30 min post-cosyntropin serum cortisol concentration of ≤18 µg/dL. No clinical manifestations of adrenal insufficiency were reported, and HPA-axis function returned to normal in all affected participants approximately 4 weeks after treatment discontinuation. The prescribing information does not require routine HPA-axis testing during standard treatment; however, clinicians should be aware that systemic absorption may be increased by application to large surface areas, prolonged use, or occlusive dressings. If HPA-axis suppression is identified, withdrawal of clascoterone should be considered [19,45]. Clascoterone 1% cream is also characterized by a favorable local safety profile, with a split-face study demonstrating no negative impact on the epidermal barrier and even showing improvement in skin hydration [27].

### 5.3. Atypical Adverse Events and Case Reports

It is important to note the potential risk of perioral dermatitis as a rare adverse effect of clascoterone therapy. A recently published case report described a 25-year-old female patient with acne associated with polycystic ovary syndrome (PCOS) and a complex medical history. After seven weeks of twice-daily application of topical clascoterone, the patient developed new-onset perioral dermatitis. The condition resolved only after the discontinuation of clascoterone and the initiation of treatment with topical tacrolimus and low-dose isotretinoin [46]. Another case involved a 23-year-old patient who developed a severe acne flare-up and facial edema three weeks after initiating topical clascoterone 1% for mild post-contraceptive acne. The reaction, characterized by painful cystic and pustular lesions, required immediate discontinuation of the drug. Clinical resolution was achieved through the use of a benzoyl peroxide cleansing bar and adapalene 0.1% [47].

In both reported cases (Table 1), discontinuation of clascoterone was necessary, and resolution was achieved through alternative topical therapies. Despite the potential for atypical adverse events in some patients, taken together, available data suggest that clascoterone is generally well tolerated across different patient populations.

The summary of published clinical studies evaluating clascoterone for acne vulgaris is presented in Table 2.

## 6. Therapeutic Positioning and Access Considerations

Current US guidelines include clascoterone as a conditionally recommended treatment option for acne vulgaris, whereas topical retinoids and benzoyl peroxide receive strong recommendations based on a stronger overall assessment of the evidence base and cost-effectiveness considerations [13]. Cost and access may further influence its clinical positioning. The Canadian Agency for Drugs and Technologies in Health did not recommend public reimbursement of clascoterone at the submitted price because the available evidence did not demonstrate sufficient cost-effectiveness [20]. Consequently, although clascoterone offers a novel mechanism of action, its uptake in routine clinical practice is likely to depend on local reimbursement policies, treatment costs, availability, and patient preferences.

## 7. Emerging Indications and Future Directions

Clascoterone has emerged as a promising candidate for the treatment of androgenetic alopecia (AGA). DHT plays an essential role in the development of AGA, and clascoterone, by virtue of its local antiandrogen activity, may be beneficial in the treatment of androgen-mediated hair loss [48]. In phase 2 studies, clascoterone treatment showed favorable outcomes in measures such as scalp sebometric measurement, hair diameter, follicular density, and pull test/wash test [49]. Clascoterone showed a hair-growth signal of comparable magnitude to that reported for minoxidil; however, no head-to-head comparison was performed [48,49].

The pivotal phase 3 program for AGA (SCALP1, NCT05910450; SCALP2, NCT05914805), described by the sponsor as the largest phase 3 clinical program conducted to date for a topical AGA therapy, enrolled 1465 men across 51 study centers in the United States and Europe and was reported to be completed in 2025. However, at the time of writing, the complete results of these trials have not been published in peer-reviewed journals and are available only through sponsor-issued announcements [50,51]. The available data should therefore be interpreted with caution pending publication of the full datasets and independent peer review.

According to the sponsor’s December 2025 announcement, both trials reportedly met their primary endpoint, with statistically significant improvements (*p* < 0.05) in Target-Area Hair Count compared with vehicle and a safety and tolerability profile described as comparable to vehicle; the reported relative improvement was 539% in SCALP1 and 168% in SCALP2 [50]. However, the large discrepancy between two identically designed trials raises concerns that require careful explanation. Twelve-month follow-up data, communicated by the sponsor in April 2026, were reported to show sustained efficacy, with continuous clascoterone treatment yielding a 2.39-fold greater improvement compared with switching to vehicle after 6 months, alongside a 24.5% relative improvement in treatment satisfaction; no significant systemic endocrine effects were reported [51]. The manufacturer has indicated that the full phase 3 dataset will be submitted for publication in a peer-reviewed journal and that regulatory submissions to the U.S. Food and Drug Administration and the European Medicines Agency for the AGA indication are anticipated. Because these findings have not undergone independent scientific evaluation, they cannot be presented as established evidence.

Until these data are independently published and reviewed, conclusions regarding the magnitude of efficacy and the long-term risk-benefit profile of clascoterone 5% solution in male AGA must remain preliminary.

Additionally, clascoterone has shown promise in the treatment of mild hidradenitis suppurativa (HS). Hargis et al. reported that at a 12-week follow-up, 10 out of 12 patients who used clascoterone cream noticed an improvement in perilesional erythema, the frequency of flares, and size of lesions. All patients who were prescribed clascoterone had stable HS or were not receiving any treatment but had residual nodular disease [40]. Cunningham et al. reported that a patient with a 5-year history of Hurley stage 1 HS also experienced improvement after clascoterone treatment [52].

Combining oral isotretinoin and topical clascoterone 1% cream may be beneficial in treating severe and/or nodular acne. Notably, clascoterone can improve facial xerosis—a frequent adverse effect of isotretinoin [41].

Several additional registered clinical trials are investigating new applications of clascoterone or further characterizing its mechanism of action (Table 3). These trials explore its potential utility in conditions ranging from acneiform rosacea and pilonidal sinus disease to steroid-related acne in transgender patients receiving masculinizing hormone therapy, as well as histologic and lipidomic evaluation of sebaceous gland effects, highlighting the broad interest in exploiting clascoterone’s antiandrogen properties across multiple dermatological conditions.

## 8. Limitations

Several limitations of the available evidence should be acknowledged. Most clinical data on clascoterone originate from industry-sponsored trials, which may introduce sponsorship, publication, and reporting bias. Moreover, independent confirmatory trials remain unavailable, limiting the external validation of the reported efficacy and safety findings. Long-term real-world evidence is also scarce, particularly regarding sustained effectiveness, adherence, uncommon adverse events, and use in patient populations that may be underrepresented in clinical trials. Published head-to-head comparisons with established acne treatments are limited, and the available network meta-analyses rely on indirect comparisons between studies with heterogeneous designs, populations, outcome definitions, and treatment durations. Therefore, conclusions regarding the relative efficacy of clascoterone should be interpreted cautiously.

The available evidence supporting emerging indications is even less certain. Data concerning androgenetic alopecia are derived predominantly from sponsor-issued announcements that have not undergone peer review, while evidence regarding hidradenitis suppurativa is limited to small observational studies and case reports. Finally, cost and reimbursement may substantially affect access to treatment and its positioning in clinical practice. These limitations reduce the certainty and generalizability of the present conclusions and underscore the need for independent comparative trials and long-term post-marketing studies.

## 9. Conclusions

Clascoterone 1% cream provides a topical approach to androgen receptor inhibition and is effective in reducing inflammatory and non-inflammatory lesions in patients with acne vulgaris. Its predominantly local adverse-event profile and suitability for both male and female patients may offer an advantage when systemic hormonal treatment is inappropriate or undesirable. Nevertheless, its relative position among established acne therapies remains incompletely defined because independent head-to-head trials and long-term real-world data are limited. Evidence concerning its use in androgenetic alopecia, hidradenitis suppurativa, and other emerging indications remains preliminary and requires confirmation in peer-reviewed, independently conducted studies.

## Figures and Tables

**Table 1:** Summary of atypical adverse events from published case reports.

Feature	Case 1 [46]	Case 2 [47]
Patient Profile	25-year-old female with PCOS and a complex medical history	23-year-old female with mild post-contraceptive acne
Treatment Regimen	Topical clascoterone 1% applied twice daily	Topical clascoterone 1% applied once daily
Onset of Symptoms	After 7 weeks of regular therapy	After 3 weeks of therapy
Diagnosis/Reaction	Perioral dermatitis	Severe acne flare-up and facial edema
Clinical Presentation	New-onset erythematous papules in the perioral area	Painful, pruritic, cystic, and pustular lesions with marked inflammation and induration

**Table 2:** The summary of published clinical studies evaluating clascoterone for acne vulgaris.

Study	Study Design	Treatment Regimen	Duration	Main Efficacy Findings	Safety Findings
Hebert et al., 2020 [18]	Randomized, double-blind	Clascoterone 1% cream BID	12 weeks	Significant improvement in IGA success and greater reduction in inflammatory, non-inflammatory and total lesion counts versus vehicle	Mainly mild local skin reactions; safety comparable to vehicle
Hebert et al., 2023 [26]	Integrated Phase III efficacy analysis	Clascoterone 1% cream BID	12 weeks	Confirmed significant efficacy across pooled Phase III studies	Favorable safety profile
Eichenfield et al., 2020 [17]	Open-label extension	Clascoterone 1% cream BID	9 months	Sustained clinical improvement throughout treatment	No new safety signals; predominantly mild adverse events
Eichenfield et al., 2023 [28]	Integrated short- and long-term analysis	Clascoterone 1% cream BID	Up to 12 months	Continued improvement with prolonged treatment	Consistent long-term safety
Draelos et al., 2025 [27]	Prospective clinical study	Clascoterone 1% cream BID	12 weeks	Sebum production decreased by 27%; inflammatory lesions by 54%; non-inflammatory lesions by 34%	Excellent tolerability; no treatment-related adverse events
Tay & Loo, 2025 [30]	Case series	Clascoterone 1% (mono-, adjunctive or maintenance therapy)	Variable	Effective in routine clinical practice; >50% continued therapy beyond 3 months	Well tolerated
Yu et al., 2025 [35]	Prospective pilot study	Clascoterone 1% BID + clindamycin/adapalene/BPO QD	8 weeks	Combination therapy showed clinical improvement	Mild burning and erythema only; no new safety concerns
Libecco et al., 2026 [37]	Subgroup analysis	Clascoterone 1% cream BID	12 weeks	Exploratory post hoc analysis: greater lesion-count reductions in adults; adjusted IGA success did not differ significantly between age groups.	Comparable safety across age and sex groups
Choi et al., 2025 [41]	Retrospective study	Oral isotretinoin ± topical clascoterone	Variable	Combination therapy associated with improved clinical outcomes	Good safety profile

**Table 3:** Summary of ongoing and currently recruiting registered clinical trials of clascoterone.

Clinical Indication	Trial ID	Phase	Enrollment	Estimated Completion	Source
Facial Acneiform Rosacea	NCT06952517	Phase 2	20	30 September 2025	[53]
Acne Vulgaris	NCT06403501	Phase 3	692	October 2026	[54]
Pilonidal Sinus Disease	NCT06286397	Phase 2	75	1 January 2028	[55]
Sebaceous gland histology (mechanistic study)	NCT06425900	Phase 4	10	March 2026	[56]
Steroid-related Acne Vulgaris in Transgender Male Patients	NCT05891795	Phase 1/2	18	June 2026	[57]

## Data Availability

No new data were created or analyzed in this study. Data sharing is not applicable to this article.

## Abbreviations

The following abbreviations are used in this manuscript:

AGA	Androgenetic alopecia
AR	Androgen receptor
BID	Bis in die (twice daily)
DHT	Dihydrotestosterone
FDA	U.S. Food and Drug Administration
HPA	Hypothalamic–pituitary–adrenal
HS	Hidradenitis suppurativa
IGA	Investigator’s Global Assessment
PCOS	Polycystic ovary syndrome
QD	Quaque die (once daily)
TEAE	Treatment-emergent adverse event

## References

[1] Williams H.C., Dellavalle R.P., Garner S. (2012). Acne vulgaris. Lancet.

[2] Kakpovbia E.E., Young T., Milam E.C., Qian Y., Yassin S., Nicholson J., Hu J., Troxel A.B., Nagler A.R. (2025). Efficacy of topical treatments for mild-to-moderate acne: A systematic review and meta-analysis of randomized control trials. J. Eur. Acad. Dermatol. Venereol..

[3] Bhate K., Williams H.C. (2013). Epidemiology of acne vulgaris. Br. J. Dermatol..

[4] Rademaker M., Garioch J.J., Simpson N.B. (1989). Acne in schoolchildren: No longer a concern for dermatologists. Br. Med. J..

[5] White G.M. (1998). Recent findings in the epidemiologic evidence, classification, and subtypes of acne vulgaris. J. Am. Acad. Dermatol..

[6] Dessinioti C., Katsambas A.D. (2010). The role of Propionibacterium acnes in acne pathogenesis: Facts and controversies. Clin. Dermatol..

[7] Adebamowo C.A., Spiegelman D., Berkey C.S., Danby F.W., Rockett H.H., Colditz G.A., Willett W.C., Holmes M.D. (2008). Milk consumption and acne in teenaged boys. J. Am. Acad. Dermatol..

[8] Wei B., Pang Y., Zhu H., Qu L., Xiao T., Wei H., Chen H., He C. (2010). The epidemiology of adolescent acne in North East China. J. Eur. Acad. Dermatol. Venereol..

[9] Haider A., Mamdani M., Shaw J.C., Alter D.A., Shear N.H. (2006). Socioeconomic status influences care of patients with acne in Ontario, Canada. J. Am. Acad. Dermatol..

[10] Choi J.M., Lew V.K., Kimball A.B. (2006). A single-blinded, randomized, controlled clinical trial evaluating the effect of face washing on acne vulgaris. Pediatr. Dermatol..

[11] Schafer T., Nienhaus A., Vieluf D., Berger J., Ring J. (2001). Epidemiology of acne in the general population: The risk of smoking. Br. J. Dermatol..

[12] Hughes H., Brown B.W., Lawlis G.F., Fulton J.E. (1983). Treatment of acne vulgaris by biofeedback relaxation and cognitive imagery. J. Psychosom. Res..

[13] Reynolds R.V., Yeung H., Cheng C.E., Cook-Bolden F., Desai S.R., Druby K.M., Freeman E.E., Keri J.E., Gold L.F.S., Tan J.K. (2024). Guidelines of care for the management of acne vulgaris. J. Am. Acad. Dermatol..

[14] Mavranezouli I., Daly C.H., Welton N.J., Deshpande S., Berg L., Bromham N., Arnold S., Phillippo D.M., Wilcock J., Xu J. (2022). A systematic review and network meta-analysis of topical pharmacological, oral pharmacological, physical and combined treatments for acne vulgaris. Br. J. Dermatol..

[15] Dhillon S. (2020). Clascoterone: First Approval. Drugs.

[16] Ferraboschi P., Legnani L., Celasco G., Moro L., Ragonesi L., Colombo D. (2014). A full conformational characterization of antiandrogen cortexolone-17α-propionate and related compounds through theoretical calculations and nuclear magnetic resonance spectroscopy. MedChemComm.

[17] Eichenfield L., Hebert A., Gold L.S., Cartwright M., Fragasso E., Moro L., Mazzetti A. (2020). Open-label, long-term extension study to evaluate the safety of clascoterone cream, 1% twice daily, in patients with acne vulgaris. J. Am. Acad. Dermatol..

[18] Hebert A., Thiboutot D., Gold L.S., Cartwright M., Gerloni M., Fragasso E., Mazzetti A. (2020). Efficacy and Safety of Topical Clascoterone Cream, 1%, for Treatment in Patients with Facial Acne: Two Phase 3 Randomized Clinical Trials. JAMA Dermatol..

[19] U.S. Food and Drug Administration (2020). WINLEVI (Clascoterone) Cream, 1% Prescribing Information. https://www.accessdata.fda.gov/drugsatfda_docs/label/2020/213433s000lbl.pdf.

[20] Balado-Simó P., Brufau-Cochs M., Morgado-Carrasco D. (2025). Clascoterone 1% in the Treatment of Acne: A Review of its Efficacy, Safety, and Therapeutic Positioning. Actas Dermosifiliogr..

[21] Del Rosso J.Q., Baldwin H., Layton A.M. (2026). Overview of the Efficacy and Safety of Topical Hormonal Therapies for the Treatment of Acne Vulgaris: A Narrative Review. J. Clin. Aesthet. Dermatol..

[22] Shaw J.C. (2002). Acne. Am. J. Clin. Dermatol..

[23] Dart D.A. (2014). Androgens have forgotten and emerging roles outside of their reproductive functions, with implications for diseases and disorders. Austin J. Endocrinol..

[24] Lai J.-J., Chang P., Lai K.-P., Chen L., Chang C. (2012). The role of androgen and androgen receptor in skin-related disorders. Arch. Dermatol. Res..

[25] Manjaly C., Martinez J., Barbieri J., Mostaghimi A. (2023). Clascoterone for treatment of acne. Drugs Today.

[26] Hebert A., Eichenfield L., Thiboutot D., Gold L.S., Vassileva S., Mihaylova Y., Cartwright M., Moro L., Fragasso E., Han J. (2023). Efficacy and Safety of 1% Clascoterone Cream in Patients Aged ≥ 12 Years with Acne Vulgaris. J. Drugs Dermatol..

[27] Draelos Z.D., Kyeremateng K., Squittieri N. (2025). Reduction in Facial Sebum Production Following Treatment with Clascoterone Cream 1% in Patients with Acne Vulgaris: 12-Week Interim Analysis. Dermatol. Ther..

[28] Eichenfield L.F., Hebert A.A., Gold L.S., Cartwright M., Moro L., Han J., Squittieri N., Mazzetti A. (2023). Long-Term Safety and Efficacy of Twice-Daily Topical Clascoterone Cream 1% in Patients ≥12 Years of Age With Acne Vulgaris. J. Drugs Dermatol..

[29] Eichenfield L.F., Gold L.S., Han J., Hebert A.A., Mazzetti A., Moro L., Squittieri N., Thiboutot D. (2024). Integrated Short-Term and Long-Term Efficacy of Topical Clascoterone Cream 1% in Patients Aged 12 Years or Older with Acne Vulgaris. J. Drugs Dermatol..

[30] Tay E., Loo W.J. (2025). Real-World Experience of Clascoterone Cream 1% in Acne Management: Case Series and Canadian Experience. Clin. Cosmet. Investig. Dermatol..

[31] Smith C.A., Gosnell E., Karatas T.B., Deitelzweig C., Collins E.M.B., Yeung H. (2025). Hormonal Therapies for Acne: A Comprehensive Update for Dermatologists. Dermatol. Ther..

[32] Basendwh M.A., Alharbi A.A., Bukhamsin S.A., Abdulwahab R.A., Alaboud S.A. (2024). The efficacy of Topical Clascoterone versus systematic spironolactone for treatment of acne vulgaris: A systematic review and network meta-analysis. PLoS ONE.

[33] Shergill M., Ali M.U., Abu-Hilal M. (2024). Comparison of the Efficacy of Clascoterone, Trifarotene, and Tazarotene for the Treatment of Acne: A Systematic Literature Review and Meta-Analysis. Dermatol. Ther..

[34] Alkhodaidi S.T., Al Hawsawi K.A., Alkhudaidi I.T., Magzoub D., Abu-Zaid A. (2021). Efficacy and safety of topical clascoterone cream for treatment of acne vulgaris: A systematic review and meta-analysis of randomized placebo-controlled trials. Dermatol. Ther..

[35] Yu Z., Swenson K., Graber E. (2025). Prospective Pilot Evaluation of the Safety, Tolerability, and Efficacy of Clindamycin Phosphate 1.2%/Adapalene 0.15%/Benzoyl Peroxide 3.1% Gel plus Clascoterone 1% Cream in Adult Patients with Acne. J. Clin. Aesthet. Dermatol..

[36] Loo W.J. (2025). Clascoterone Cream 1% in Acne Management: Case Series and Real-World Canadian Experience. Can. Dermatol. Today.

[37] Libecco J., Eichenfield L.F., Hebert A.A., Gold L.S., Cartwright M., Moro L., Sane S., Lionnet L., Shukla D., Mazzetti A. (2026). Efficacy and Safety of Clascoterone Cream 1% for Acne Are Independent of Age and Sex. J. Drugs Dermatol..

[38] Dréno B., Thiboutot D., Gollnick H., Finlay A.Y., Layton A., Leyden J.J., Leutenegger E., Perez M. (2010). Large-scale worldwide observational study of adherence with acne therapy. Int. J. Dermatol..

[39] Rosette C., Agan F.J., Mazzetti A., Moro L., Gerloni M. (2019). Cortexolone 17α-propionate (clascoterone) is a novel androgen receptor antagonist that inhibits production of lipids and inflammatory cytokines from sebocytes in vitro. J. Drugs Dermatol..

[40] Hargis A., Yaghi M., Maskan Bermudez N., Lev-Tov H. (2024). Clascoterone in the treatment of mild hidradenitis suppurativa. J. Am. Acad. Dermatol..

[41] Choi U., Xiong G., Metko D., Cy A., Pathayappura S., Bawazir M., Abu-Hilal M. (2025). Efficacy of oral isotretinoin monotherapy compared to combination isotretinoin and topical clascoterone for severe acne vulgaris: A multi-center retrospective study. J. Dermatol. Treat..

[42] Täubel J., Mazzetti A., Ferber G., Burch W., Fernandes S., Patel A., Spencer C.S., Freier A., Graff C., Kanters J.K. (2021). A Phase 1 Study to Investigate the Effects of Cortexolone 17α-Propionate, Also Known as Clascoterone, on the QT Interval Using the Meal Effect to Demonstrate ECG Assay Sensitivity. Clin. Pharmacol. Drug Dev..

[43] Del Rosso J., Stein Gold L., Squittieri N., Thiboutot D. (2023). Is There a Clinically Relevant Risk of Hyperkalemia with Topical Clascoterone Treatment?. J. Clin. Aesthet. Dermatol..

[44] Di Stefano A.F.D., Radicioni M.M., Garhöfer G., Cartwright M., Müller M., Kopeloff I., Moro L., Squittieri N., Mazzetti A. (2025). Pharmacokinetics, Safety, and Skin Irritation and Sensitization Potential of Clascoterone Cream in Early-Phase Clinical Study Participants. Clin. Pharmacol. Drug Dev..

[45] Bhatia N., Eichenfield L.F., Mazzetti A., Moro L., Squittieri N., Hebert A.A. (2024). Hypothalamic-Pituitary-Adrenal Axis Response in Patients with Acne Vulgaris Treated with Clascoterone. J. Drugs Dermatol..

[46] Noda M., Zureigat M. (2026). Perioral dermatitis secondary to topical clascoterone 1%. JAAD Case Rep..

[47] Castillo A.L., Rojas R.F., Garavito S.J.C., Muñoz M.C.A. (2026). Severe acne induced by clascoterone: A case report. JAAD Case Rep..

[48] Sun H.Y., Sebaratnam D.F. (2020). Clascoterone as a novel treatment for androgenetic alopecia. Clin. Exp. Dermatol..

[49] Mazzetti A., Moro L., Gerloni M., Cartwright M. (2019). A summary of in vitro, phase I, and phase II studies evaluating the mechanism of action, safety, and efficacy of clascoterone in androgenetic alopecia. J. Cosmet. Dermatol. Sci. Appl..

[50] Cosmo Pharmaceuticals N.V. (2025). Cosmo announces breakthrough Phase III topline results from SCALP 1 and SCALP 2 for clascoterone 5% solution in male hair loss, showing up to 539% relative improvement in Target-Area Hair Count versus placebo. Press Release. https://www.cosmohealthconfidence.com/news/11029858-breezula-phase-iii-results.

[51] Cosmo Pharmaceuticals N.V. (2026). Phase III 12-Month Data for Clascoterone 5% Topical Solution Confirm Positive Safety for Chronic Use and Continued Hair Growth. Press Release. https://www.cosmohealthconfidence.com/news/98958067-clascoterone-12-month-safety-results-ende.

[52] Cunningham K.N., Moody K., Alorainy M., Rosmarin D. (2023). Use of topical clascoterone for the treatment of hidradenitis suppurativa. JAAD Case Rep..

[53] Narrows Institute for Biomedical Research Efficacy and Safety of Topical Clascoterone Cream 1% in Patients with Facial Acneiform Rosacea. ClinicalTrials.gov Identifier: NCT06952517. NCT06952517.

[54] Zhejiang Sunshine Mandi Pharmaceutical Co., Ltd Efficacy and Safety of Clascoterone Cream 1% in Treatment of Subjects with Facial Acne Vulgaris. ClinicalTrials.gov Identifier: NCT06403501. NCT06403501.

[55] University of Pennsylvania Topical Anti-Androgens in Pilonidal Sinus Disease. ClinicalTrials.gov Identifier: NCT06286397. NCT06286397.

[56] Sun Pharmaceutical Industries Limited Histologic and Liquid Chromatography Mass Spectrometry (LCMS) Evaluation of the Sebaceous Gland Changes Induced by Clascoterone Cream 1%. ClinicalTrials.gov Identifier: NCT06425900. NCT06425900.

[57] Chang A. Topical Androgen Receptor Inhibitor for Steroid-related Acne Vulgaris in Transgender Male Patients Receiving Masculinizing Hormone Therapy. ClinicalTrials.gov Identifier: NCT05891795. NCT05891795.

